# Dietary Long-Chain Fatty Acids Accelerate Metabolic Dysfunction in Guinea Pigs with Non-Alcoholic Steatohepatitis

**DOI:** 10.3390/nu15112445

**Published:** 2023-05-24

**Authors:** Kamilla Pedersen, David Højland Ipsen, Josephine Skat-Rørdam, Jens Lykkesfeldt, Pernille Tveden-Nyborg

**Affiliations:** 1 Section of Experimental Animal Models, Department of Veterinary and Animal Sciences, Faculty of Health and Medical Sciences, University of Copenhagen, 1870 Frederiksberg, Denmark; kamilla.pedersen@sund.ku.dk (K.P.); dvie@novonordisk.com (D.H.I.); jsr@sund.ku.dk (J.S.-R.); jopl@sund.ku.dk (J.L.); 2 Integrated Physiology Research, Obesity and NASH Pharmacology, Novo Nordisk A/S, 2760 Måløv, Denmark

**Keywords:** non-alcoholic fatty liver disease (NAFLD), non-alcoholic steatohepatitis (NASH), medium-chain fatty acid, long-chain fatty acid, glucose intolerance, uric acid, guinea pigs

## Abstract

The composition of dietary fatty acids may be important for the development and progression of metabolic syndrome and non-alcoholic steatohepatitis (NASH). This study investigated the effect of two high-fat diets based on coconut oil, containing predominantly medium-chain fatty acids (MCFA), or cocoa butter, containing mainly long-chain fatty acids (LCFA), on glucose homeostasis and NASH in guinea pigs following 16 and 32 weeks of diet. At week 16, glucose intolerance was increased in the LCFA animals compared to the MCFA animals (*p* < 0.001), with both groups differing from the controls by week 32 (*p* < 0.0001), supported by increased hemoglobin A1c (*p* < 0.05). NASH was present in both high-fat groups from week 16, with advancing fibrosis appearing more progressive in the LCFA animals at week 16. In agreement, gene expression showed overall increased expression of NASH target genes in the LCFA animals compared to the MCFA animals at weeks 16 and 32 (*p* < 0.05 and *p* < 0.0001, respectively). The LCFA animals also displayed increased plasma uric acid at both time points (*p* < 0.05), a phenomenon linked to NASH in humans. In conclusion, this study reports that a diet high in LCFA promotes metabolic imbalance and may accelerate NASH-associated hepatic fibrosis. This highlights the importance of a critical evaluation of fatty acid composition when investigating NASH-associated endpoints.

## 1. Introduction

The prevalence of non-alcoholic fatty liver disease (NAFLD) has increased rapidly during the last few decades, mirroring the escalating number of obesity and type 2 diabetes cases, and is currently estimated to affect about 25% of the global adult population [1]. Most commonly driven by excess intake of dietary fat, cholesterol, and simple sugars, NAFLD can be considered the hepatic manifestation of metabolic syndrome [2]. The term covers a wide spectrum of hepatic conditions from simple steatosis to non-alcoholic steatohepatitis (NASH) and may progress to cirrhosis and/or hepatocellular carcinoma [3]. Reflective of metabolic overload, the main risk factor for developing NAFLD is an unhealthy lifestyle combined with an inadequate level of physical activity [4].

The source of dietary fat has been suggested to play an important role in the development and progression of NAFLD [5]. The different effects of dietary fat are attributed both to the length and degree of saturation of the fatty acids, with the length of fatty acids commonly categorized as short- (<8 carbons), medium- (8–12 carbons), or long-chain (>12 carbons) fatty acids [5,6]. Medium-chain fatty acids (MCFAs) are typically found in dairy products and coconut oil [7], whereas long-chain fatty acids (LCFAs) are dominant in various fat sources both from plants and animals [8]. Studies in experimental animals have shown that diets rich in LCFAs increase liver steatosis, glucose intolerance, body fat, and oxidative stress compared with diets rich in MCFAs [9,10,11,12,13,14,15]. However, the findings across studies are not entirely congruent, as some studies have reported that diets rich in LCFAs led to decreased body fat deposition and insulin resistance compared with diets rich in MCFAs [12,15]. Though controlled studies investigating the effects of fatty acid chain length in humans are scarce, LCFAs—as opposed to MCFAs—have been found to increase liver steatosis and body fat [16,17,18]. 

LCFAs may be saturated or have one (mono-unsaturated) or more (poly-unsaturated) double bonds in their carbon chain, with the degree of saturation clearly altering their chemical and biological properties [10,19,20]. While omega-3 poly-unsaturated LCFAs in particular are reported to attenuate NAFLD and other aspects of metabolic syndrome [9,10,21], the effect of mono-unsaturated LCFAs is more ambiguous: When compared with MCFAs, some animal studies find that mono-unsaturated LCFAs increase steatosis and glucose intolerance [9,10,19,22], whereas others report similar or decreased steatosis and glucose intolerance [23,24]. Patients with NASH have increased plasma levels of both saturated and mono-unsaturated LCFAs [25], and a recent cross-sectional study in NAFLD/NASH patients has strongly supported fatty acid composition as key in NASH pathogenesis and disease severity in humans [26]. 

The aim of the present study was to compare how two different fat sources, one based on coconut oil (rich in MCFAs) and one based on cocoa butter (rich in LCFAs), affect the development of NASH and metabolic imbalance measured by glucose homeostasis in the guinea pig NASH model [27,28,29,30]. The findings were compared to a parallel lean control group [30]. The results support that a cocoa-butter-based LCFA diet accelerates glucose intolerance and likely NASH severity, specifically the development of fibrosis and triglyceride accumulation, compared to a coconut-oil-based MCFA diet.

## 2. Materials and Methods

### 2.1. Animals and Experimental Design

The animal experiments were conducted in accordance with Directive 2010/63/EU and approved by the Danish Animal Experiments Inspectorate (license no. 2018-15-0201-01591) and are reported according to ARRIVE guidelines [31]. Forty-eight female Hartley guinea pigs (Charles River Laboratories, Lyon, France) weighing 300–350 g were subcutaneously microchipped (E-vet, Haderslev, Denmark) upon arrival. They were group-housed in floor pens with wood shavings, hay, straw, shelters, and environmental enrichment under a 12 h light–dark cycle with temperatures between 20–24 ℃. The animals were inspected daily, and their bodyweights were recorded weekly. 

**Figure 1:**
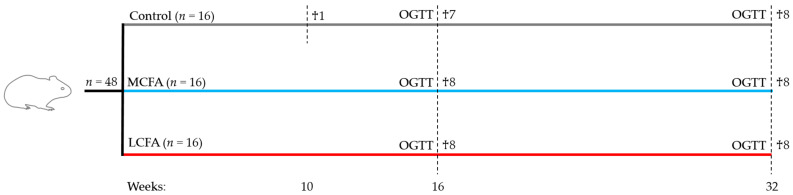
Study design. Forty-eight guinea pigs were block-randomized based on weight into three different diet groups: a low-fat control diet (control), a MCFA-rich high-fat diet (MCFA), and a LCFA-rich high-fat diet (LCFA). They had one week of acclimatization. One guinea pig in the control group was not included as it was euthanized during week 10 due to exceeding the humane endpoint of 20% lower bodyweight than the group mean. Seven animals from the control group and eight animals from the MCFA and LCFA groups were euthanized (♰) after 16 weeks. These animals underwent an oral glucose tolerance test (OGTT) prior to euthanization. The remaining animals continued on the diet for 32 weeks and underwent OGTT before euthanization.

**Figure 2:**
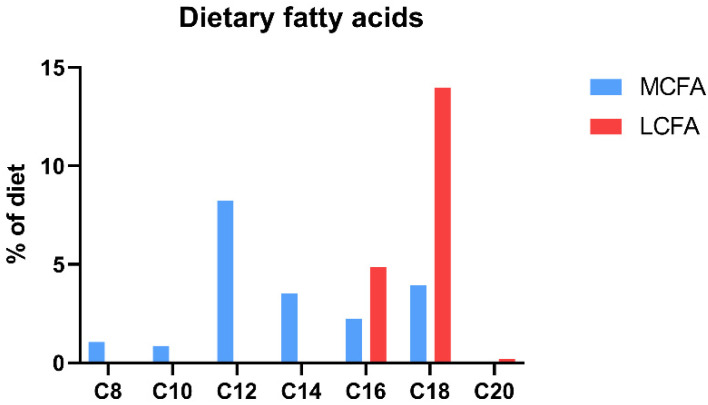
Dietary fatty acid distribution. The graph illustrates the different lengths of fatty acids in the two high-fat diets. MCFA: medium-chain fatty-acid-rich high-fat diet (coconut oil) and LCFA: long-chain fatty-acid-rich high-fat diet (cocoa butter).

The animals were block-randomized based on weight into three groups, receiving different chow-based diets: (1) a low-fat control diet (control, *n* = 16); (2) a medium-chain fatty-acid-rich high-fat diet (MCFA, *n* = 16); and (3) a long-chain fatty-acid-rich high-fat diet (LCFA, *n* = 16) (Ssniff Spezialdiäten GmbH, Soest, Germany) (Study overview: Figure 1; diet composition: Table 1; fatty acid composition: Table 2; dietary fatty acid distribution: Figure 2). The data on the animals allocated to the control and MCFA diets have previously been published as part of a parallel intervention study conducted simultaneously [32]. The studies were conducted in parallel to reduce the number of animals according to the 3R principles of the experimental practice of using live animals. The group sizes were based on power calculations with a power of 0.8 and a significance level of <0.05. Variances were based on our previous studies, and an effect size of 30% between means of selected endpoints (adhering to NASH) was considered biologically relevant. All animals were fed a standard guinea pig diet during the first week of acclimatization, followed by a gradually increasing titration with the respective experimental diets over the course of 5 days. The feed intake in each group was estimated twice weekly by weighing the feed remains before feeding. To ensure that the control group was lean and metabolically healthy, animals were pair-fed to match two-thirds of the equivalent calories ingested by the MCFA group. The feed was stored at −20 °C and thawed daily to reduce auto-oxidation and maintain palatability. One guinea pig in the control group was euthanized during week 10 due to weight loss exceeding the humane endpoint of a maximum of 20% lower body weight compared with the control group mean, leaving 15 animals in the control group. The necropsy of this animal did not show any signs of underlying disease.

Following 16 weeks on the diet, seven animals from the control group and eight animals from each high-fat group were randomly selected for euthanization. The remaining animals continued on the diet for a total of 32 weeks (Figure 1). Prior to termination, the animals were subjected to an oral glucose tolerance test (OGTT) (see Section 2.2. for details). Upon study termination, the animals were block randomized for euthanization over the course of five days to avoid potential time-dependent bias and as previously described [27,33]. In short, the guinea pigs were semi-fasted overnight (i.e., access to hay but not feed) before being pre-anesthetized with 1.25 mL/kg body weight 10-fold diluted Zoletil mixture (125 mg tiletamin and 125 mg zolazapam (Zoletil 50 vet; Virbac, Nice, France) supplemented with 200 mg xylazine (Narcoxyl vet 20 mg/mL; Intervet International, Boxmeer, Holland) and 7.5 mg butorphanol (Torbugesic vet 10 mg/mL; Scanvet, Fredensborg, Denmark)) and placed on inhalation anesthesia with 3–5% isoflurane (Isoba vet 100%, Intervet International, Denmark) after 15–20 min [27]. Once completely anesthetized, an intracardial blood sample was collected followed by euthanization by decapitation.

### 2.2. Oral Glucose Tolerance Test (OGTT)

Prior to euthanization, the animals underwent an OGTT (Figure 1). The OGTTs were performed over the course of three days, with the order of animals determined by weight-stratified randomization. The guinea pigs were semi-fasted (allowed access to hay but not feed) for 16 h prior to testing as described previously [30]. Blood glucose was measured in duplicates or triplicates with a glucometer (Accu-Check Aviva, Roche Diagnostics, Bern, Switzerland) by the punctuation of an auricular vein with a 27 G needle [34]. A baseline concentration was measured, followed by oral dosing of 2 g/kg BW of a 150% glucose solution (0.7–1.3 mL depending on body weight). The glucose solution was administered through a syringe placed in one side of the animal’s mouth and subsequent voluntary ingestion, while gently fixating the guinea pig. Blood glucose was measured at 30, 60, 90, and 120 min after glucose administration. 

### 2.3. Plasma Samples

Blood samples for plasma analyses were collected as intracardial samples on anesthetized animals immediately prior to euthanization. All analyses of plasma samples were performed on single samples in a randomized and blinded manner and as described below.

Blood samples for analysis of ascorbate and uric acid were collected in 10 mL syringes flushed with K_3_-EDTA [35]. The samples were centrifuged at 2000× *g* for four minutes at 4 °C followed by the collection of plasma. The plasma was stabilized in 10% meta-phosphoric acid (MPA) and stored at −80 °C until analysis by electrochemical detection through high-performance liquid chromatography (HPLC) as previously described [36].

Blood samples for the analysis of aspartate aminotransferase (AST), alanine aminotransferase (ALT), triglycerides (TG), and total cholesterol (TC) were collected in K3 EDTA coated microvettes (Sarstedt, Nümbrecht, Germany). Samples for analyses of alkaline phosphatase (ALP) and free fatty acids (FFA) were collected in heparin- and sodium-fluoride-coated microvettes (Sarstedt, Nümbrecht, Germany), respectively. The samples were centrifuged at 2000× *g* for four minutes at 4 °C followed by the collection of plasma into Cobas cups (Sample cup micro 13/16, Roche Diagnostics, Mannheim, Germany). Intracardial blood was also collected in 10 µL Na-Heparinized End-to-End Vitrex^®^ Pipettes (Vitrex medical A/S, Herlev, Denmark) for analysis of hemoglobin A1c (HbA1c). These samples were mixed with 1 mL Hemolyzing Reagent nr. 11488457 122 (Cobas, Roche diagnostics, Rotkreuz, Switzerland) and left on ice to incubate for 10 min. A maximum of 200 µL was moved to a Cobas cup. All of the plasma samples in the Cobas cups were stored at −20 °C until analysis according to the manufacturer’s instructions on a Cobas 6000 (Roche Diagnostic Systems, Bern, Switzerland). 

### 2.4. Liver Samples

Immediately after euthanization, the liver was carefully removed, rinsed in cold phosphate-buffered saline (PBS) (140 mM NaCl, 10 mM phosphate, 3 mM KCl, pH 7.4, Millipore, Billerica, MA, USA), weighed, and photographed. Samples for histology were cut into 3–5 mm-thick slices from the left lateral liver lobe and fixed in 10% formalin. From the same liver lobe, 3–5 mm-thick slices were either frozen on dry ice for biochemical analyses or snap-frozen in liquid nitrogen for gene expression analyses and stored at −80 °C. 

Biochemical analysis of ascorbate and uric acid was performed by HPLC as previously described [37]. In short, a 100–200 mg liver sample was homogenized in cold Dulbecco’s PBS (D8537, Sigma-Aldrich, Darmstadt, Germany), stabilized in 10% MPA-EDTA, and centrifuged at 16,000× *g* for one minute at 4 °C. The supernatant was stored at −80 °C until analysis on HPLC. Biochemical analyses of TGs and TC were performed on a Cobas 6000 according to the manufacturer’s instructions as previously reported [27,30]. In short, 1.0 mL extraction buffer (0.15 M sodium acetate and 0.75% Triton-X) was added to a 30–40 mg frozen tissue sample followed by homogenization. The homogenates were placed in boiling water for two minutes and then cooled on ice and supplemented with 0.5 mL extraction buffer. Subsequently, a 500 µL sample was centrifuged at 9000× *g* for 10 min at 4 °C. The collected supernatants were then analyzed using a Cobas 6000. All biochemical analyses on the liver homogenate were performed on single samples in a randomized and blinded manner.

### 2.5. Histology

The formalin-fixed liver samples were embedded in paraffin, before sectioning (2–4 µm thick) and stained with hematoxylin and eosin (H&E) or picrosirius red (PSR). Scoring reproducibility was verified by calculation of Cohen’s kappa index as described previously [30]. The histological sections were evaluated in a blinded and randomized manner, and semi-quantitatively scored in accordance with the scoring scheme suggested by Kleiner et al. [38] and applied to the guinea pig NASH model with a few modifications [27,28]. In short, the scoring criteria were as follows: steatosis, lobular inflammation, and ballooning hepatocytes were scored on the H&E stains. Steatosis was evaluated across the entire slide at ×5 or ×10 magnification and graded 0 (<5%), 1 (<5–33%), 2 (>33–66%), or 3 (>66%) based on the distribution of intracellular lipid droplets in the hepatocytes. Hepatocyte lipid depositions were confirmed in this model with oil red O staining in a previous study [27]. Lobular inflammation was assessed at ×20 magnification in six lobules (two portal areas at a similar distance to a central vein) counting the number of inflammatory foci (≥3 inflammatory cells in close proximity) and scored as 0 (none), 1 (1 focus), 2 (2–4 foci), or 3 (>4 foci). The degree of ballooning hepatocytes was evaluated across the entire liver section at ×40 magnification as 0 (no ballooning hepatocytes), 1 (a few ballooning hepatocytes), or 2 (many/prominent ballooning hepatocytes). To assess the overall disease severity, the NAFLD activity score (NAS) was calculated as the unweighted sum of steatosis, lobular inflammation, and ballooning hepatocytes scores ranging from zero to eight [38]. Fibrosis was evaluated on PSR stained sections as F0 (not present), F1 (A: mild zone 3/perisinosoidal, B: moderate zone 3/perisinosoidal, and C: portal/periportal), F2 (perisinosoidal and portal/periportal), F3 (bridging from central vein to central vein, from central vein to portal vein, and/or from portal vein to portal vein), and F4 (A: mild cirrhosis, B: moderate cirrhosis, and C: severe cirrhosis) [39]. The amount of fibrosis in relation to the total amount of liver tissue was quantified using Visiopharm software (version 2020.08.4.9377, VisioPharm, Hørsholm, Denmark) as previously described [32]. 

### 2.6. Gene Expression

RNA extraction, reverse transcription, and qPCR were performed as previously reported [29]. In short, 50 mg of liver from each animal was homogenized in 1000 µL MagMax Lysis/Binding Solution Concentrate (Thermo Fisher, Waltham, MA, USA) with 0.7% β-mercaptoethanol (Sigma Aldrich, St. Louis, MO, USA). The samples were centrifuged for 1 min at 10,000 RPM and 4 °C, the supernatant was isolated and stored at −20 °C for 24 h, followed by RNA isolation using a MagMax-96 Total RNA Isolation Kit (Thermo Fisher, Waltham, MA, USA). To synthesize the cDNA, 500 ng RNA was subjected to reverse transcription (High Capacity cDNA Reverse Transcription Kit; Thermo Fisher, Waltham, MA, USA) on a 2720 Thermal Cycler (Applied Biosystems, Foster City, CA, USA) under the following conditions: 25 °C for 10 min, 37 °C for 120 min, and 85 °C for 5 s. All cDNA samples were confirmed free of genomic DNA contamination using an intron-spanning primer set (β-actin, Table 2) before inclusion [40].

qPCR analysis was performed by adding 2 µL of 3 ng/µL cDNA to 8 µL Master Mix (5 µL PowerUp SYBR Green Master Mix (Thermo Fisher, Waltham, MA, USA), 1 µL Primer mix, and 2 µL RNAse-free water). All of the samples were run in triplicate on the StepOnePlusTM Real Time PCR system (Applied Biosystems, Foster City, CA, USA) under the following conditions: 50 °C for 2 min, 95 °C for 5 min, and then 40 cycles consisting of 95 °C for 10 s, 60 °C for 10 s, and 72 °C for 20 s. Melting curves were evaluated for each primer set to confirm the product specificity. The primer sequences were either acquired from previously published sequences [29] or designed with NCBI Primer-BLAST or Primer3 (Table 3). The new primer products were sequenced (Eurofins, Ebersberg, Germany) and their target specificity was confirmed by NCBI BLAST sequence alignment. Dynactin subunit 5 (*DCTN5*) displayed homogenous expression between groups and was selected as the reference gene. 

### 2.7. Statistics

The statistical analyses were performed by using GraphPad version 9.4.1 (GraphPad Prism software, La Jolla, CA, USA). Continuous normally distributed data with equal variances between groups were analyzed by one-way ANOVA, two-way ANOVA, or a mixed-effects model (in case of missing values), with repeated measures when relevant, and Tukey’s test for multiple comparisons, and the results are presented as the means with the standard deviation (SD). If the data deviated from a Gaussian distribution, they were transformed (by log or square root), re-analyzed, and presented as medians with 25th and 75th percentiles. If the data continued to deviate from normality or in the case of categorical data, they were analyzed as non-parametric data with a Kruskal–Wallis’ test and Dunn’s test for multiple comparisons and presented as medians with 25th and 75th percentiles. Data with unequal variances between groups were analyzed by Welch’s test with Dunnett’s test for multiple comparisons and presented as medians with 25th and 75th percentiles. The qPCR data were analyzed by the ∆∆CT method and presented as fold-changes with ranges [45]. The ∆∆CT values and their standard deviations for each target gene were analyzed by two-way ANOVA with Tukey’s test for multiple comparisons. To evaluate putative overall effects in gene expression patterns in the two high-fat groups (MCFA and LCFA), a two-way ANOVA of all of the target genes’ CT values and standard deviations was applied at weeks 16 and 32.

## 3. Results

### 3.1. Body Weights and Liver Size

**Figure 3:**
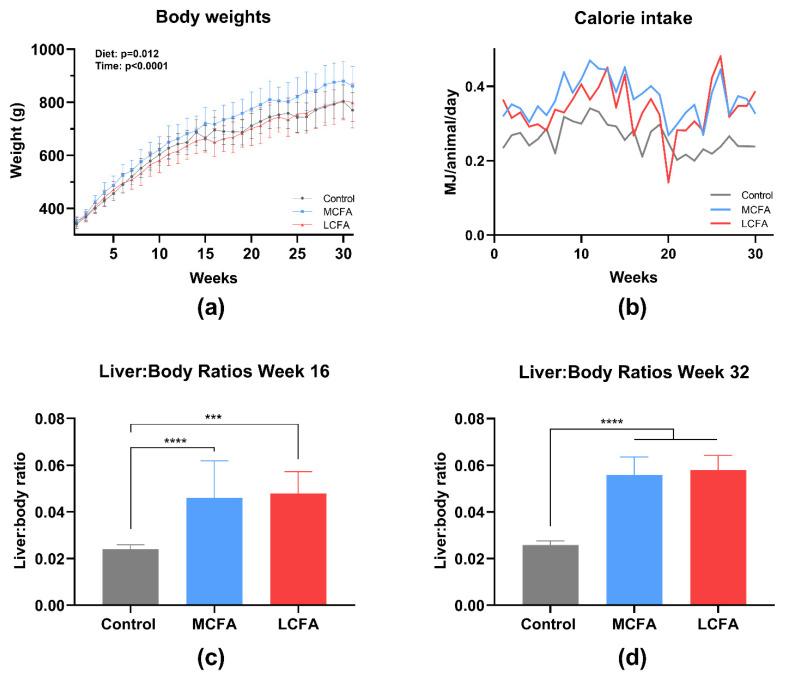
Body weights, feed intake, and liver:body ratios. (**a**) Body weights across the experimental period; the log-transformed data were analyzed using a mixed-effects model with repeated measures. The overall effects of diet and time are shown on the graph. The data are presented as the means with the SD. (**b**) Calorie intake across the experimental period estimated pr. animal from the group feed intake. The MCFA and LCFA groups were fed ad libitum, while the control group was pair-fed to ~67% of the equivalent calories ingested by the MCFA group. The data were analyzed with a Mann–Whitney test and are presented as exact values. (**c**) The liver:body ratios at week 16 and (**d**) the liver:body ratios at week 32. The log-transformed liver:body ratios were analyzed by Welch’s test with Dunnett’s multiple comparisons test and are presented as medians with the inter-quartile range. *** *p* < 0.001 and **** *p* < 0.0001. The body weights for the control and MCFA groups have been published previously [32]. Control: low-fat control diet, MCFA: medium-chain fatty-acid-rich high-fat diet, and LCFA: long-chain fatty-acid-rich high-fat diet.

When analyzing across the entire experimental period, the effect of diet was significant (*p* = 0.012) with the MCFA animals weighing more than the LCFA and the control animals (control: 666 g ± 47 g and 771 g ± 67 g; MCFA: 720 g ± 60 g and 861 g ± 75 g; LCFA: 662 g ± 50 g and 798 g ± 71 g; weeks 15 and 31, respectively) (Figure 3a). The two high-fat diets were isocaloric. Hence, the significant difference between the body weights in terms of LCFA and MCFA was unexpected; however, this was likely explained by an overall 8.5% lower calorie intake in the LCFA animals compared with MCFA based on the area under the curve (AUC) of the caloric intake throughout the study period (Figure 3b). The mean AUC (± standard error of the mean) differed between the groups (control: 7.60 ± 0.13; MCFA: 10.73 ± 0.20; LCFA: 9.82 ± 0.26), with the control AUC being smaller than both MCFA and LCFA (*p* < 0.0001) and LCFA being smaller than MCFA (*p* = 0.008). Despite weighing and eating less, the liver-to-body ratios were similar between LCFA and MCFA at both euthanasia time points (Figure 3c,d). 

### 3.2. Oral Glucose Tolerance Test (OGTT)

**Figure 4:**
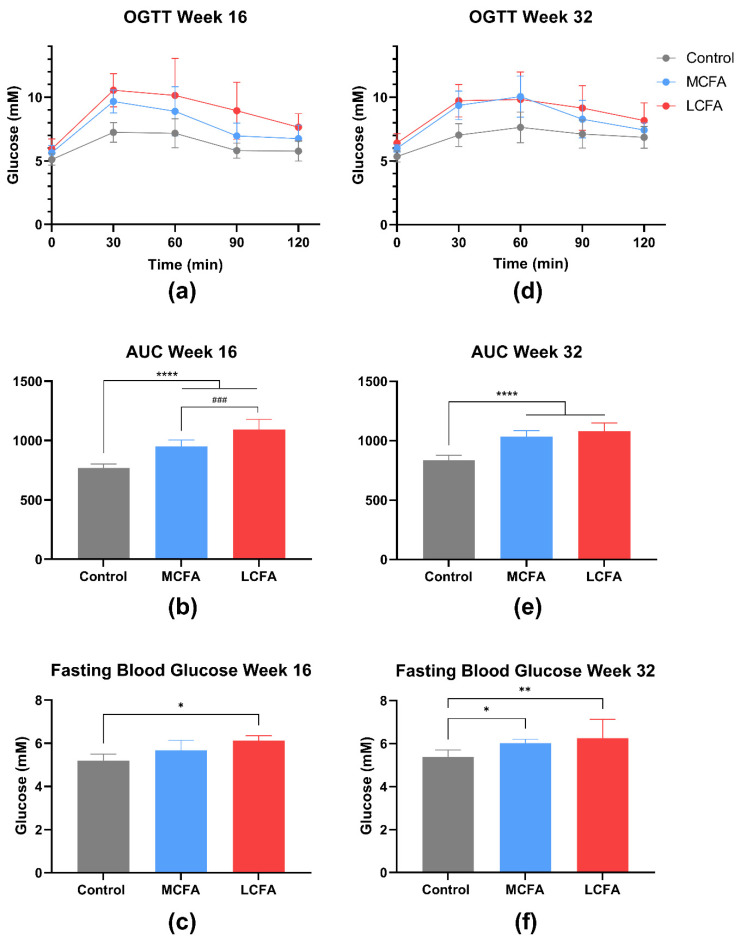
Oral glucose tolerance tests (OGTTs). The OGTT curves and the area under the curve (AUC) are presented as the means with the SD. The fasting blood glucose is presented as the medians with the inter-quartile range. (**a**) The OGTT curve at week 16. (**b**) The AUC at week 16; the data were analyzed by a one-way ANOVA with Tukey’s multiple comparisons test. (**c**) The fasting blood glucose at week 16; the data were analyzed by a Kruskal–Wallis test with Dunn’s multiple comparisons test. (**d**) The OGTT curve at week 32. (**e**) The AUC at week 32; the data were analyzed by a one-way ANOVA with Tukey’s multiple comparisons test. (**f**) The fasting blood glucose at week 32; the data were analyzed by a Kruskal–Wallis test with Dunn’s multiple comparisons test. * *p* < 0.05, ** *p* < 0.01, and **** *p* < 0.0001, compared to the control group. ^###^ *p* < 0.001, compared to the MCFA group. The data (except the fasting blood glucose) for the control and MCFA groups have been published previously [32]. Control: low-fat control diet, MCFA: medium-chain fatty-acid-rich high-fat diet, and LCFA: long-chain fatty-acid-rich high-fat diet.

To assess the effect of the different diets on glucose homeostasis as a measure of the metabolic state of the animals, OGTTs were performed before euthanasia. At week 16, the AUC for the LCFA and MCFA groups was significantly higher than the control animals (*p* < 0.0001), and the AUC of the LCFA animals was also significantly higher than the MCFA animals (*p* < 0.001) (Figure 4a,b). If normalized to the baseline values, the difference in the AUCs persisted (data not shown). The LCFA group showed significantly higher fasting glucose levels compared with the control group at week 16 (*p* = 0.045) (Figure 4c), supporting that glucose homeostasis was affected. No difference was detected between the groups when comparing the HbA1c plasma levels at week 16 (Table 4).

At week 32, the LCFA and MCFA animals displayed higher AUCs compared to the control animals (*p* < 0.0001), whereas no difference between the OGTT AUCs was detected between the LCFA and MCFA groups (Figure 4d,e). Both the MCFA group and the LCFA group displayed significantly increased fasting glucose compared to the control animals (*p* = 0.048 and *p* = 0.008, respectively) (Figure 4f). The level of fasting blood glucose was not significantly different between the LCFA group and the MCFA group (Figure 4f). At week 32, HbA1c was significantly higher in both high-fat groups compared with the control group (MCFA: *p* = 0.018; LCFA: *p* = 0.015) (Table 4), supporting altered glucose tolerance induced by the long-term high-fat dietary regimes.

### 3.3. Plasma Biochemistry

The plasma lipid profile was measured by FFA, TG, and TC (Table 4). At both week 16 and week 32, the LCFA animals had significantly higher plasma FFA (*p* = 0.025 and *p* = 0.037, respectively), and at week 32, they had lower TG concentrations (*p* = 0.030) compared with the control group. This was not the case for the MCFA animals (Table 4). As expected, both high-fat groups had markedly higher concentrations of TC compared to the control group; however, the MCFA group showed significantly higher TC concentrations than the LCFA group at both week 16 and week 32 (*p* = 0.008 and *p* = 0.001, respectively).

Liver health was assessed by measuring the plasma levels of AST, ALT, and ALP (Table 4). AST was significantly increased in the high-fat groups compared with the control group at both week 16 (MCFA: *p* = 0.011; LCFA: *p* = 0.012) and week 32 (*p* < 0.0001), whereas ALT was significantly increased at week 32 (MCFA: *p* = 0.042; LCFA: *p* = 0.002). No difference was present in ALP at week 16, but at week 32, the MCFA group showed a significantly lower ALP level than the control group (*p* = 0.044).

**Figure 5:**
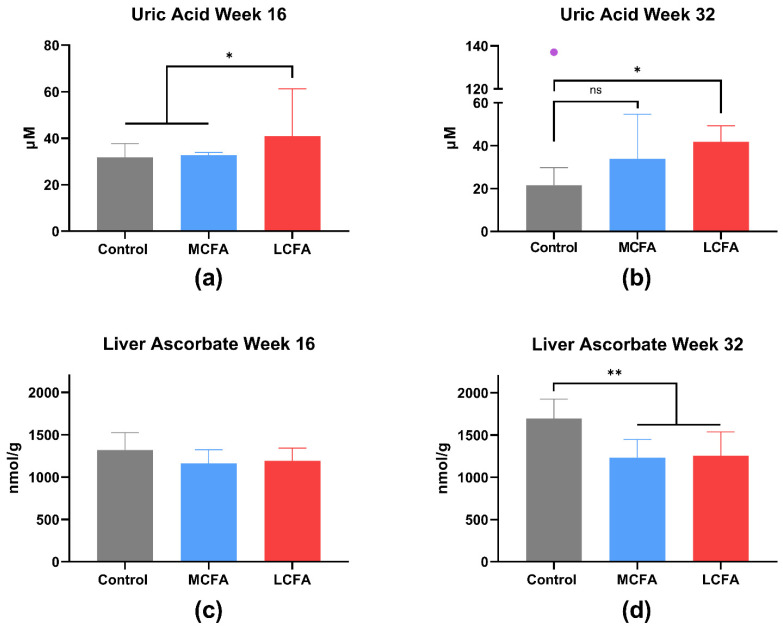
Uric acid and liver ascorbate. Uric acid is presented as the medians with the inter-quartile range. Liver ascorbate is presented as the means with SD. (**a**) Uric acid in plasma at week 16. The log-transformed data were analyzed by a one-way ANOVA with Tukey’s multiple comparisons test. (**b**) Uric acid in plasma at week 32. The data were analyzed by a Kruskal–Wallis test with Dunn’s multiple comparisons test. An outlier (the purple point) from the control group was recorded and subsequently not included in the statistical test; if the outlier was included, there was no difference between the three groups. (**c**,**d**) Liver ascorbate at week 16 and week 32; the data were analyzed by a one-way ANOVA with Tukey’s multiple comparisons test. * *p* < 0.05 and ** *p* < 0.01. Control: low-fat control diet, MCFA: medium-chain fatty-acid-rich high-fat diet, and LCFA: long-chain fatty-acid-rich high-fat diet.

The concentration of uric acid in plasma was significantly higher in the LCFA group compared to the control group at both time points (week 16: *p* = 0.023; week 32: *p* = 0.018) and compared to the MCFA group at week 16 (*p* = 0.033) (Figure 5a,b). In one animal from the control group, the uric acid concentration was more than double the highest measured values in any other of the measured samples; hence, it was excluded from the statistical analysis. The ascorbate levels in plasma did not differ between the groups (Table 4).

### 3.4. Liver Biochemistry

To evaluate the lipid load in the liver, TGs and TC were measured. The liver TG levels in the high-fat groups were significantly higher than in the control group at both week 16 (MCFA: *p* = 0.03; LCFA: *p* < 0.001) and week 32 (*p* < 0.0001). At week 32, the LCFA group had significantly higher TG content than the MCFA group (*p* < 0.0001). Interestingly, the TG levels in both the LCFA group and the MCFA group decreased at week 32 (Table 5). As seen in the plasma, the liver TC was significantly increased in the LCFA group and the MCFA group compared with the control group at both time points (*p* < 0.0001). The MCFA group had significantly more liver TC than the LCFA group at week 16 (*p* = 0.042) but not by week 32 (Table 5). Liver ascorbate was not different between the groups at week 16, but both the MCFA group and the LCFA group had significantly less ascorbate in their livers than the control group at week 32 (*p* = 0.003 and *p* = 0.005, respectively) (Figure 5c,d).

### 3.5. Histopathology

**Figure 6:**
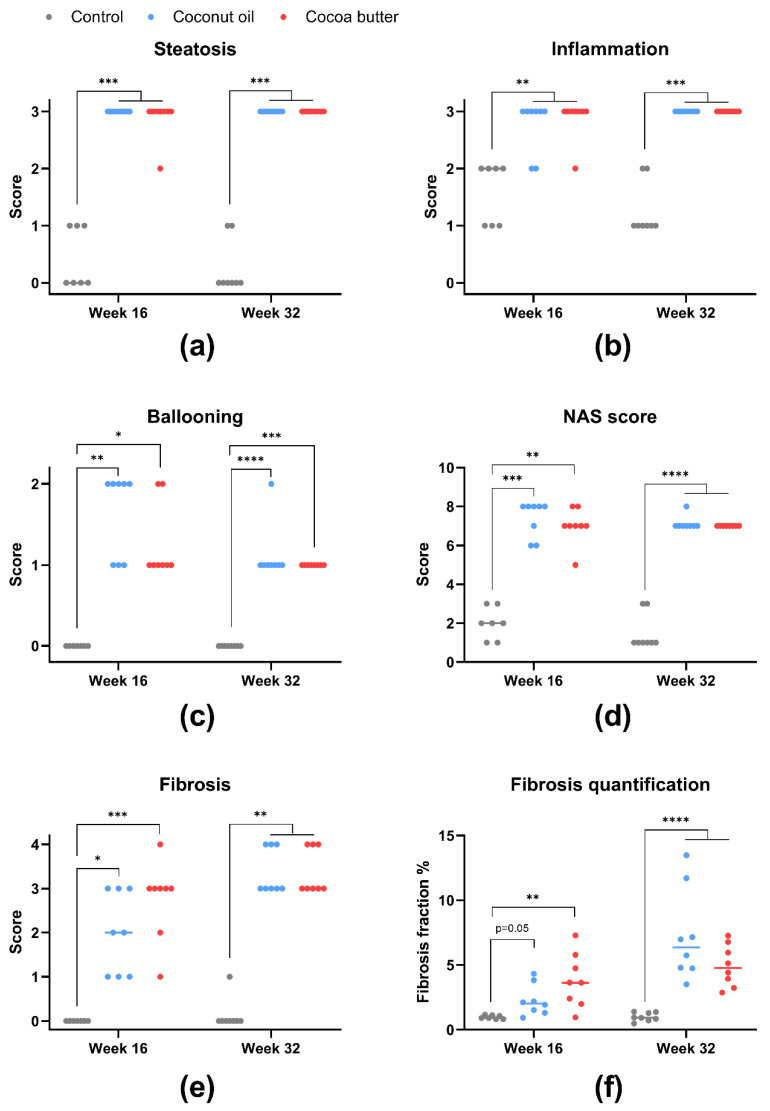
Histopathological scoring and fibrosis quantification. The data are presented as the individual scores with the medians. (**a**) The steatosis scores on a scale of 0–3, (**b**) the inflammation scores on a scale of 0–3, (**c**) the ballooning scores on a scale of 0–2, (**d**) the NAFLD activity score on a scale of 0–8, (**e**) the fibrosis scores on a scale of 0–3, and (**f**) fibrosis quantification in %. The histopathological scores and fibrosis fraction at week 16 were analyzed using the non-parametric Kruskal–Wallis test with a Dunn’s multiple comparisons test. The log-transformed fibrosis fraction at week 32 was analyzed by one-way ANOVA. * *p* < 0.05, ** *p* < 0.01, *** *p* < 0.001, and **** *p* < 0.0001. The data for the control and MCFA groups have been published previously [32]. Control: low-fat control diet, MCFA: medium-chain fatty-acid-rich high-fat diet, and LCFA: long-chain fatty-acid-rich high-fat diet.

**Figure 7:**
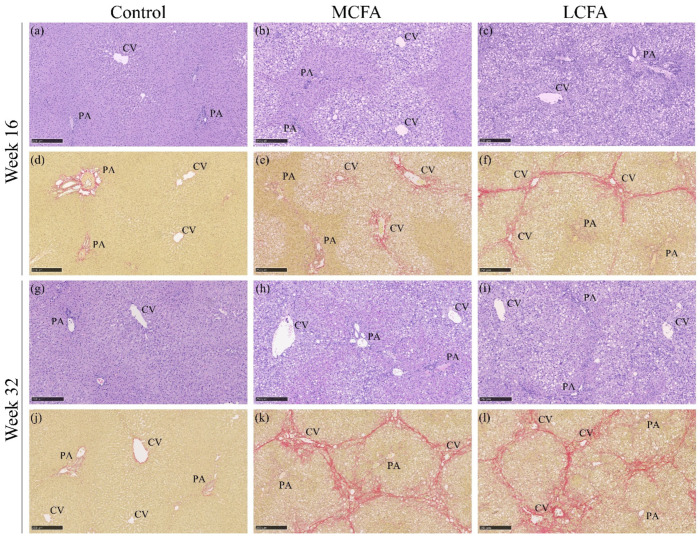
Representative histological images from each group at both time points. (**a**–**c**) Hematoxylin and eosin stain at week 16, (**d**–**f**) picrosirius red stain at week 16, (**g**–**i**) hematoxylin and eosin stain at week 32, (**j**–**l**) picrosirius red stain at week 32. (**k**,**l**) show livers with F4A scores (mild cirrhosis). The scale bar shows 250 µm. CV: central vein; PA: portal area. Control: low-fat control diet, MCFA: medium-chain fatty-acid-rich high-fat diet, and LCFA: long-chain fatty-acid-rich high-fat diet.

To evaluate the degree of liver damage induced by the high-fat diets, histopathology was assessed and scored (Figure 6 and Figure 7). The MCFA and LCFA groups showed severe steatosis, inflammation, and ballooning at both time points (Figure 6a–c). None of the variables showed any difference between the LCFA and MCFA groups, although the MCFA group tended to have slightly higher ballooning scores than the LCFA group at week 16 (Figure 6c). This was also reflected in the NAFLD activity score (NAS) (Figure 6d). At week 16, both high-fat groups had significantly more fibrosis compared with the control group (MCFA: *p* = 0.012; LCFA: *p* < 0.001) (Figure 6e). The LCFA group had a median fibrosis score of 3, whereas the MCFA group had a median fibrosis score of 2. Quantification of fibrosis area in the liver sections showed a significant difference between the LCFA group and the control group (*p* = 0.002) but no significant difference between the MCFA group and the control group (*p* = 0.050) (Figure 6f). Surprisingly, one LCFA animal developed mild cirrhosis (F4a) after only 16 weeks on the diet, a finding that our group has not previously reported when using an MCFA-rich diet to induce this model. By week 32, the LCFA and MCFA groups had reached similar levels of fibrosis, reflected in both the fibrosis score and the quantification (Figure 6e,f). At this time point, three animals with cirrhosis (fibrosis grade F4) were evident in both high-fat-fed groups (MCFA: F4A = one animal and F4B = two animals; LCFA: F4A = three animals). Together, the data demonstrate that the LCFA-diet and MCFA-diet both nduced NASH with severe fibrosis and progressing to cirrhosis suggest that hepatic fibrosis may occur somewhat earlier in animals receiving an LCFA diet.

### 3.6. Gene Expression

**Figure 8:**
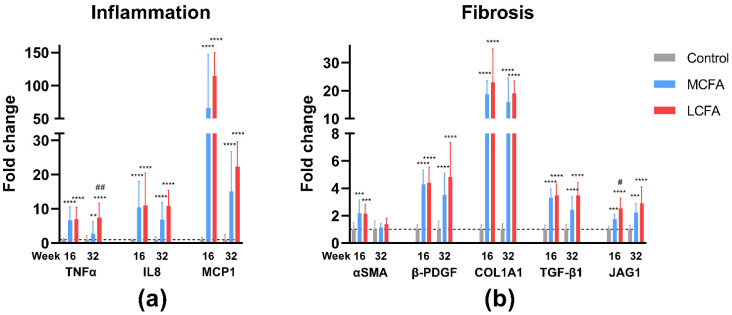
Expression of inflammatory and fibrotic genes. The data were analyzed by two-way ANOVA with Tukey’s multiple comparisons test and are presented as the means with ranges expressed as fold changes relative to the control. (**a**) Expression of inflammatory genes: TNFα, tumor necrosis factor α; IL8, interleukin 8; MCP1, monocyte chemotactic protein 1. (**b**) Expression of fibrotic genes: αSMA, α smooth muscle actin; β-PDGF, β-platelet-derived growth factor; Col1a1, collagen 1a1; TGF-β1, transforming growth factor β1; JAG1, jagged1. Different from the control group ** *p* < 0.01, different from the control group *** *p* < 0.001, different from the control group **** *p* < 0.0001. Different from the MCFA group # *p* < 0.05; different from the MCFA group ## *p* < 0.01. Control: low-fat control diet, MCFA: medium-chain fatty-acid-rich high-fat diet, and LCFA: long-chain fatty-acid-rich high-fat diet.

To elaborate on the putative cellular mechanisms affected by the diets and linked to the progression of NASH, the expression of key inflammatory and fibrotic genes was analyzed. As expected, both the MCFA group and the LCFA group showed significantly higher expression of inflammatory and fibrotic genes compared with the control group (Figure 8). After 16 weeks on the diet, the LCFA group showed significantly higher expression of jagged canonical Notch ligand 1 (JAG1), a fibrogenic and tumorigenic gene that is overexpressed in patients with advanced NASH [46,47], compared with the MCFA group (*p* = 0.029). At week 32, the LCFA group showed significantly higher expression of the pro-inflammatory cytokine tumor necrosis factor α (TNFα) (*p* = 0.005). When comparing the two high-fat groups with a two-way ANOVA of all of the investigated target genes, the LCFA group had overall increased gene expression at both week 16 and week 32 (*p* = 0.030 and *p* < 0.0001, respectively). 

## 4. Discussion

This study reports that in a guinea pig model, a cocoa-butter-based diet rich in LCFAs accelerated the development of glucose intolerance—a key component of the metabolic syndrome—compared with a diet rich in MCFAs (coconut-oil-based). Furthermore, the LCFA diet increased liver TG content and plasma uric acid and appears to have accelerated NASH-associated liver fibrosis. 

Both high-fat diets affected glucose homeostasis at week 16 and week 32. However, the LCFA diet induced more severe glucose intolerance at week 16 than the MCFA diet, as measured by fasting blood glucose concentrations and AUC. This finding is consistent with studies in other animal models comparing the effects of MCFA and LCFA diets on glucose homeostasis and confirms the relevance of fatty acid composition in the etiology of metabolic dysregulation across different species [10,13,14,15,48]. In addition, MCFA-induced beneficial effects on glucose homeostasis have also been reported in humans [49,50]. It may be speculated that glucose uptake in the skeletal muscles could be part of an underlying mechanism associated with this response. The skeletal muscles are responsible for >80% of glucose uptake and are considered a primary driver of whole-body insulin resistance [51]. Insulin resistance is strongly associated with intramyocellular triglyceride content [52]. As MCFAs have been shown to induce increased upregulation of oxidation capacity in striated muscles compared to LCFAs [14,48], it could be speculated that muscle cells may clear the dietary lipid oversupply, thereby preventing myocellular lipid accumulation and hence insulin resistance. However, the pathophysiological impact of this suggested mechanism remains to be investigated.

In agreement with findings in humans and experimental animal models, the LCFA diet promoted hepatic steatosis compared with the MCFA diet [9,10,11,18]. The increased steatosis may be explained by the higher levels of LCFAs from the cocoa butter displaying a greater affinity for being stored as intracellular TGs for secretion and subsequent transport to peripheral storage sites [53] as opposed to MCFAs that are more efficiently oxidized [6,54]. TGs are secreted from the liver through very low-density lipoproteins (VLDL), but VLDL assembly and secretion are limited by the apoB100 and MTTP proteins. While lipid export from hepatocytes is initially increased in NAFLD, the export stagnates—or even decreases—as the disease progresses, resulting in advancing steatosis [55]. A significant decrease in plasma TGs was present in the LCFA group at week 32, supporting that lipid export from the liver was compromised in the LCFA group compared to the MCFA group, thereby posing a possible mechanism for the observed difference in plasma TG levels. TG accumulation per se may not be harmful to the hepatocytes [56,57], but depleting the capacity of VLDL assembly and secretion will likely upregulate LCFA oxidation. Whereas MCFAs can pass mitochondrial membranes freely, LCFAs depend on transport proteins to enter the mitochondria, consequently limiting the β-oxidation of LCFAs to the available transport capacity [6]. If the transport capacity is exceeded, it will lead to the accumulation of FFAs that are cytotoxic [58,59]. Furthermore, β-oxidation of LCFAs produces more reactive oxygen species (ROS) than β-oxidation of MCFAs [54], likely leading to earlier development of oxidative stress as the hepatocyte antioxidant capacity is exceeded. Collectively, an overload of LCFAs may therefore be expected to induce liver damage faster than an overload of MCFAs. This is reflected by the seemingly accelerated liver fibrosis in the LCFA group at week 16, supporting that NASH advances to a more severe disease stage earlier in animals subjected to a diet high in LCFAs from cocoa butter than in animals receiving the MCFA (coconut oil) diet.

Interestingly, uric acid levels were higher in the LCFA group at both time points and in the MCFA animals at week 32 compared to the healthy controls. Previous studies have found a link between NAFLD or NASH and hyperuricemia in patients [60,61,62,63], though the underlying mechanisms are still unclear. Uric acid is the end-product of purine metabolism, primarily derived from the degradation of cellular nucleic acids [64]. It is possible that the increased cell turnover due to liver damage is a source of hyperuricemia in NASH. Studies have shown that uric acid induces hepatic fat accumulation, oxidative stress, and activation of the NLRP3 inflammasome, linking uric acid to steatosis and inflammation [65,66]. Hepatic ascorbate levels in the LCFA and MCFA groups compared to the age-matched controls were significantly reduced at week 32 but not at week 16. Seemingly, the high-fat groups did not show an increase in hepatic ascorbate levels from week 16 to week 32 as the control group did. The control group was pair-fed with the MCFA group, ensuring equal vitamin C intake, but the LCFA group consumed 8.5% less feed over the experimental period than the MCFA group. Thus, we expected the LCFA group to have lower ascorbate levels than the MCFA group, but this was not the case. However, the two high-fat groups consumed comparable quantities of feed for at least three weeks prior to both euthanasia time points, most likely explaining the similar hepatic ascorbate levels. The reduced hepatic ascorbate levels in the high-fat groups support increased antioxidant consumption, likely reflecting increased production of reactive free radicals, which is in agreement with oxidative stress as a key factor in NASH development. 

At week 32, the LCFA group displayed lower body weights compared to the MCFA group, likely due to the decreased feed intake in the LCFA animals. Studies from other animal models have not reported this effect [9,11], but it may well be due to the guinea pigs—renowned to be very picky eaters—finding cocoa butter (the LCFA diet) less palatable than coconut oil (the MCFA diet). Considering that the LCFA group consumed 8.5% fewer calories than the MCFA group and that the control group consumed 33% fewer calories than the MCFA group, we did not anticipate the control group and the LCFA group to have similar body weights. Nevertheless, guinea pigs have previously been shown to be effective caloric compensators [28,30], so it is possible that the control group consumed more hay than the high-fat groups to make up for the reduced calorie ration from the feed. Importantly, though the LCFA group consumed less feed, hence a lower amount of fat than the MCFA group, the LCFA diet still accelerated glucose intolerance, liver TG accumulation, and likely fibrosis compared to the MCFA diet. We therefore speculate that the LCFA-associated effects may have been more substantial had the feed intake been equal to the MCFA group.

Though sharing several similarities in fatty acid composition [67,68], the choice of cocoa butter over lard may limit the comparability to studies applying a lard-based diet as the LCFA source. Many animal studies investigating the effect of fatty acid chain length on NAFLD choose lard as the source of LCFAs; however, guinea pigs are strict herbivores and are dependent on hindgut fermentation for processing nutrients. Feeding lard could potentially induce a detrimental effect on the gut microbiome and digestion, compromising feed uptake and nutrition. The current study provides insights into the NASH-promoting effects of diets with different fatty acid sources containing different levels of MCFA and LCFA. However, an isolated role of specific fatty acids cannot be assessed. This is a potential limitation to the study; however, the current aim was to evaluate whether a different fatty acid source—rather than a specific fatty acid on its own—would influence NASH and NASH progression in the animal model. In this aspect, our study has shown that the fatty acid source is important, supporting that future studies of putative mechanisms should be performed, e.g., including investigations of isolated fatty acids. 

In conclusion, this study shows that a cocoa-butter-based LCFA diet accelerates metabolic dysfunction in the form of glucose intolerance and appears to accelerate NASH-associated liver fibrosis when compared to a coconut-oil-based MCFA diet. This highlights the importance of fatty acid sources as dietary drivers of NASH and the importance of critically evaluating dietary regimes in both human patients and experimental animal studies. 

## Figures and Tables

**Table 1:** Diet composition.

Content	Control	MCFA	LCFA
Product number:	S9406-S042	S9406-S0253	S9406-S050
Crude protein (%)	17.1	16.9	16.9
Crude fat (%)	3.8	20.0	20.0
Crude fiber (%)	19.8	11.4	11.4
Crude ash (%)	7.9	6.6	6.6
Starch (%)	13.4	7.9	7.9
Sugar (%)	4.0	17.3	17.3
Cholesterol (%)	0.0	0.350	0.350
ME (MJ/kg)	11.2	16.8	16.8
Ascorbic acid (mg/kg)	2000	2000	2000

The chow-based diet composition is provided by the manufacturer and is calculated based on the mean values of the ingredient analyses. Ascorbic acid is added as phosphorylated ascorbic acid (Stay-C). ME: metabolizable energy. Control: low-fat control diet, MCFA: medium-chain fatty-acid-rich high-fat diet (coconut oil), and LCFA: long-chain fatty-acid-rich high-fat diet (cocoa butter).

**Table 2:** Fatty acid composition.

Fatty Acids	Control	MCFA	LCFA
C8:0 (%)	-	1.06	-
C10:0 (%)	-	0.86	-
C12:0 (%)	-	8.22	-
C14:0 (%)	0.01	3.53	0.02
C16:0 (%)	0.64	2.23	4.80
C18:0 (%)	0.11	2.32	6.27
C20:0 (%)	0.01	0.03	0.19
C16:1 (%)	0.02	0.01	0.07
C18:1 (%)	0.67	0.44	6.13
C18:2 (%)	1.89	0.96	1.31
C18:3 (%)	0.35	0.22	0.27
C20:1 (%)	<0.01	<0.01	<0.01
Total MCFAs (%)	-	10.14	-
Total LCFAs (%)	3.70	9.74	19.06
Total saturated fatty acids (%)	0.77	18.25	11.28
Total unsaturated fatty acids (%)	2.93	1.63	7.78

The chow-based diet composition is provided by the manufacturer and is calculated based on the mean values of the ingredient analyses. C18:3 is in the form of alpha-linolenic acid. Control: low-fat control diet, MCFA: medium-chain fatty-acid-rich high-fat diet (coconut oil), and LCFA: long-chain fatty-acid-rich high-fat diet (cocoa butter).

**Table 3:** Primer sequences used for hepatic gene expression.

Gene	Accession No.	Forward	Reverse	Product (bp)
*ACTA2* (αSMA) [41]	ENSCPOT00000011693.2	GACATCAAGGAGAAGCTGTG	GCTGTTGTAGGTGGTTTCAT	273
*COL1A1* [41]	XM_003466865.2	CTGGACAGCGTGGTGTAGTC	TCCAGAAGGACCTTGTTTGC	104
*PDGFB* (β-PDGF) [29]	XM_013153075.1	CCCCTCCAGCAGATGAAGTT	GGTCTCAATCCAGGGTCCAA	199
*TGFB1* (TGF-β1) [42]	NM_001173023.1	AACCCGAGCCGGACTACTATG	TGCTTTTATAGATATTGTGGC TGTTGT	78
*CXCL8* (IL8) [43]	NM_001173399.2	GGCAGCCTTCCTGCTCTCT	CAGCTCCGAGACCAACTTTGT	67
*TNF* (TNFα) [44]	NM_001173025.1	GCCGTCTCCTACCCGGAAAA	TAGATCTGCCCGGAATCGGC	203
*CCL2* (MCP1) [29]	NM_001172926.1	TGCCAAACTGGACCAGAGAA	CGAATGTTCAAAGGCTTT GAAGT	75
*JAG1*	XM_003476557.4	ACAGGACAACAGGGACTTGG	AGTGCCCTCCGATTCTACCT	105
*ACTB* (β-ACTIN) [40]	AF508792	GTAAGGACCTCTATGCCAACACA	ATGCCAATCTCATCTCGTTTTCT	346
*DCTN5*	XM_003477819.4	TTGACGGGATTCTGAGGTGC	CACAACACTGACTGGCGACT	122

The previously published primer pairs are referenced (square brackets). αSMA, α smooth muscle actin; COL1A1, collagen 1a1; β-PDGF, β-platelet-derived growth factor; TGF-β1, transforming growth factor β1; IL8, interleukin 8; TNFα, tumor necrosis factor α; MCP1, monocyte chemotactic protein 1; JAG1, jagged canonical notch ligand 1; DCTN5, dynactin subunit 5.

**Table 4:** Plasma biochemistry.

	Week	Control	MCFA	LCFA
FFA (mmol/L)	16 ^1^	0.54 (0.49—0.62)	0.61 (0.54–0.68)	0.75 (0.59–0.91) *
	32 ^1^	0.35 (0.19–0.54)	0.59 (0.51–0.61)	0.60 (0.50–0.84) *
TG (mmol/L)	16 ^1^	0.64 (0.58–0.82)	0.70 (0.50–0.85)	0.75 (0.63–0.86)
	32 ^2^	0.65 (0.54–0.81)	0.54 (0.45–0.66)	0.52 (0.42–0.58) *
TC (mmol/L)	16 ^3^	0.52 (0.49–0.85)	7.12 (4.98–11.23) ****	3.17 (2.54–4.55) ****^,##^
	32 ^2^	0.53 (0.43–0.66)	7.42 (5.94–7.84) ****	4.24 (3.78–5.17) ****^,##^
AST (U/L)	16 ^2^	126 (30.7–329)	398 (169–1003) *	441 (243–492) *
	32 ^2^	31.9 (20.4–36.2)	470 (395–556) ****	555 (305–718) ****
ALT (U/L)	16 ^1^	29.7 (20.2–35.8)	44.0 (33.8–62.9)	44.0 (32.2–78.4)
	32 ^3^	33.0 (25.2–36.5)	66.9 (48.9–93.5) *	72.3 (57.2–98.6) **
ALP (U/L)	16 ^1^	59 (41–68)	53 (42–56)	55 (39–80)
	32 ^1^	56 (48–61)	44 (37–49) *	53 (47–57)
HbA1c (%)	16 ^1^	3.9 (3.8–4.0)	4.0 (3.9–4.2)	3.9 (3.8–4.3)
	32 ^1^	3.9 (3.8–4.0)	4.3 (4.0–4.5) *	4.2 (4.1–4.8) *
Ascorbate (µmol/L)	16 ^4^	39.1 ± 6.1	35.4 ± 10.2	39.5 ± 8.6
	32 ^4^	46.6 ± 12.2	33.0 ± 12.6	44.3 ± 14.1

The data are presented as the medians with quartiles (Q25-Q75) in brackets or means ± SD. ^1^ The data were analyzed using a Kruskal–Wallis test with Dunn’s multiple comparisons test. ^2^ The log-transformed data were analyzed by one-way ANOVA with Tukey’s multiple comparisons test. ^3^ The data were analyzed using Welch’s test with Dunnett’s multiple comparisons test. ^4^ The data were analyzed by a one-way ANOVA with Tukey’s multiple comparisons test. Different from the control group * *p* < 0.05, ** *p* < 0.01, and **** *p* < 0.0001. Different from the MCFA group ^##^ *p* < 0.01. The data (except for ascorbate) for the control and MCFA group have been published previously [32]. FFA: free fatty acids, TG: triglycerides, TC: total cholesterol, AST: aspartate aminotransferase ALT: alanine aminotransferase, ALP: alkaline phosphatase, and HbA1c: hemoglobin A1c (% glycated hemoglobin of total hemoglobin). control: low-fat control diet, MCFA: medium-chain fatty-acid-rich high-fat diet, and LCFA: long-chain fatty-acid-rich high-fat diet.

**Table 5:** Liver lipids.

	Week	Control	MCFA	LCFA
TG (µmol/g)	16 ^1^	7.06 (6.15–10.1)	55.0 (46.3–62.7) *	70.8 (55.3–75.3) ***
	32 ^2^	4.79 (4.34–5.97)	39.7 (33.4–42.5) ****	59.3 (52.6–66.9) ****^,####^
TC (µmol/g)	16 ^3^	4.31 (3.91–5.05)	32.0 (30.4–36.3) ****	28.5 (24.4–31.4) ****^,#^
	32 ^3^	4.10 (3.77–4.46)	27.3 (24.7–32.5) ****	28.3 (27.6–33.4) ****

The data are presented as the medians with the quartiles (Q25-Q75) in brackets. ^1^ The data were analyzed using a Kruskal–Wallis test with Dunn’s multiple comparisons test. ^2^ The square-root-transformed data were analyzed by one-way ANOVA with Tukey’s multiple comparisons test. ^3^ The log-transformed data were analyzed by one-way ANOVA with Tukey’s multiple comparisons test. Different from the control group * *p* < 0.05, different from the control group *** *p* < 0.001, different from the control group **** *p* < 0.0001, different from the MCFA group ^#^ *p* < 0.05, and different from the MCFA group ^####^ *p* < 0.0001. The data for the control and MCFA groups have been published previously [32]. TG: triglycerides, TC: total cholesterol, Control: low-fat control diet, MCFA: medium-chain fatty-acid-rich high-fat diet, and LCFA: long-chain fatty-acid-rich high-fat diet.

## Data Availability

The data presented in this study are available from the corresponding author upon request.
